# Moth-Eye-Inspired Antireflective Structures in Hybrid Polymers: Depth-Variable Etching Techniques, Optical Performance, Thermal Stability, and Hydrophobicity

**DOI:** 10.3390/nano15070490

**Published:** 2025-03-25

**Authors:** Lukas Werner, Zhaolu Diao, Joachim P. Spatz, Marcus Abend, Steffen Resche, Nico Hagen, Richard Busch, Robert Brunner

**Affiliations:** 1 Department SciTec, University of Applied Sciences Jena, Carl-Zeiss-Promenade 2, 07745 Jena, Germany; lukas.werner@eah-jena.de (L.W.); marcus.abend@eah-jena.de (M.A.);; 2 Institute of Nanostructure Technologies and Analytics (INA), Technological Electronics Department, University of Kassel, Heinrich-Plett-Straße 40, 34132 Kassel, Germany; 3 Max Planck Institute for Medical Research, Jahnstraße 29, 69120 Heidelberg, Germanyspatz@mr.mpg.de (J.P.S.); 4 Institute for Molecular Systems Engineering (IMSE), Heidelberg University, Im Neuenheimer Feld 246, 69120 Heidelberg, Germany; 5 Max Planck School Matter to Life, Jahnstraße 29, 69120 Heidelberg, Germany; 6 Modern Camera Designs GmbH, Moritz-von-Rohr Straße 1a, 07745 Jena, Germany; 7 Fraunhofer Institute for Microstructure of Materials and Systems (IMWS), Walter-Hülse-Straße 1, 06120 Halle, Germany; 8 Fraunhofer Institute for Applied Optics and Precision Engineering (IOF), Albert-Einstein-Straße 7, 07745 Jena, Germany

**Keywords:** antireflective structures, moth-eye nanostructures, transmission efficiency, hybrid polymers, reactive ion etching, thermal stability

## Abstract

Hybrid polymers combine the benefits of inorganic and organic material properties, offering superior thermal, mechanical, and chemical stability, making them ideal for optical applications. This study focuses on the fabrication and characterization of antireflective (AR) structures within hybrid polymers using reactive ion etching (RIE). The etching process produces nanopillars with controlled heights, achieving excellent AR performance across a broad spectral range from 450 nm to 2 µm. Optical characterization, including angle-resolved transmission and reflection measurements, shows that the structured samples maintain high transmission efficiency and reduced reflectance at varying incidence angles. Thermal stability tests reveal that the AR structures preserve their optical properties after exposure to temperatures up to 250 °C. Higher temperatures cause significant material yellowing, which is attributed to changes in the bulk material rather than damage to the structured surface. Hydrophobicity measurements show significant water repellency in structured samples, with contact angles more than twice those of unstructured layers. These findings highlight the potential of hybrid polymers with moth-eye-inspired nanostructures for high-performance, durable optical components in demanding environments.

## 1. Introduction

Hybrid polymers represent a class of composite materials in which inorganic and organic components are interconnected at the molecular level. This unique structure combines the advantageous properties of inorganic glass and organic polymers. Particularly for optical elements, hybrid polymers offer excellent characteristics such as high transparency, resistance to yellowing, and precisely tunable wavelength-dependent dispersion properties. Compared to purely polymeric optical materials, hybrid polymers exhibit superior thermal, mechanical, and chemical stability [1,2].

A notable example is the hybrid polymer ORMOCER^®^ (ORganically Modified CERamics) [3,4], which demonstrates outstanding thermal stability. Cured patterns of ORMOCER^®^ maintain structural integrity at temperatures of up to 300 °C for short-term exposure and 270 °C for long-term use. In contrast, conventional optical polymers such as polystyrene (PS) and polymethylmethacrylate (PMMA) are limited to operating temperatures of approximately 60 °C, while polycarbonate (PC) can withstand temperatures only up to 130 °C [5]. Furthermore, as UV-curable materials, hybrid polymers are particularly advantageous for the high-throughput production of optical components via efficient replication processes like nanoimprinting and UV molding. In addition to UV replication in batch processes [6,7], optical elements based on hybrid polymers can also be fabricated using additive manufacturing techniques, including modern 3D printing methods [8]. This capability facilitates both large-scale production and rapid prototyping. Due to these beneficial properties, hybrid polymers have been widely adopted in optical applications, including micro-optical arrays [6,9], particularly for structured illumination projectors in the automotive industry, such as “welcome light carpets” [10]. Other notable applications include freeform lens arrays for artificial compound eye cameras [7], microconcentrators for photovoltaic systems [11], and waveguide Bragg gratings [12].

Like all optical elements, antireflection (AR) coatings are essential for hybrid-polymer-based optical components to enhance light efficiency, suppress stray light, and improve contrast. However, conventional layer-based AR coatings have adhesion issues, which limit the utilization of hybrid polymers’ high thermal stability and restrict their performance over a wide temperature range. These issues arise from differences in thermal expansion coefficients between the coating and the substrate, potentially leading to damage of the AR coating. As an alternative, subwavelength structured elements offer a viable solution by integrating directly into the substrate material, thereby creating a continuous gradient-index transition between the surrounding medium and the substrate. Inspired by biological analogs, such structures are commonly referred to as “moth-eye structures” [13].

Artificial moth-eye structures can be fabricated in a variety of optical materials using diverse manufacturing techniques [14]. One such approach involves a two-step process, in which a mask is first created using micron- or nanoscale polystyrene spheres, followed by dry etching to generate quasiperiodic structures [15]. On glass substrates, these structures enable broadband and highly angle-tolerant antireflection properties due to their precisely controlled small feature sizes [13]. Alternative fabrication methods, particularly suited for thermoplastic polymers, involve the stochastic generation of nanostructures on polymer substrates and organic layers via low-pressure plasma etching [16,17]. However, these etching processes are typically limited to structural depths of approximately 100 nm. By applying and subsequently etching additional organic layers, deeper structures exceeding 200 nm can be achieved [18]. This approach also allows for the incorporation of AR structures into thermoplastic cycloolefin polymer optics [19]. Unfortunately, the application of additional layers can lead to adhesion problems.

Other fabrication techniques include stamping methods, wherein nanostructures are first created in a mold and subsequently replicated into either the substrate material or an overlying layer [20]. However, these replication processes generally achieve structural depths ranging from just over 100 nm to less than 300 nm. AR nanostructures have also been successfully fabricated in hybrid polymers using ultraviolet nanoimprint lithography. For instance, interference lithography stamps have been employed to transfer structures onto polyethylene terephthalate (PET) substrates, increasing optical transmission from 90% to 93% [21]. Similarly, ref. [22] describes the replication of AR structures via nanoimprint lithography, achieving average structure heights below 300 nm. To enhance the efficiency of perovskite solar cells, ref. [23] utilized a silicon stamp produced by electron-beam lithography, also attaining structure heights below 300 nm.

This paper presents the fabrication of antireflective (AR) structures in hybrid polymers using dry etching processes, particularly reactive ion etching (RIE). Unlike conventional approaches that require additional layer preparation, this method enables direct etching of the hybrid polymer substrate. By varying the etching duration, the height of stochastic pillar-like structures can be controlled, reaching maximum mean heights of up to 830 nm. Excellent antireflective performance is observed, covering a broad spectral range from the deep blue at approximately 450 nm to the infrared region up to 2 µm.

A systematic characterization of the fabricated AR structures is presented in the following sections. Structural properties are analyzed using electron microscopy, including focused ion beam (FIB) cross-sectioning, while optical performance is investigated based on transmittance and reflectance measurements across different wavelengths and angles. Furthermore, wettability is assessed through contact angle measurements, and thermal stability studies are conducted to evaluate performance under elevated temperatures.

## 2. Sample Preparation, Structural Analysis, and Initial Optical Characterization of AR-Structured Hybrid Polymer Surfaces

The hybrid polymer samples were prepared on inorganic glass substrates using a customized and proprietary UV molding process at company mcd—modern camera designs GmbH (Jena, Germany). Float glass wafers (Borofloat B33; Schott, Wolverhampton, UK) with dimensions of approximately 65 × 65 mm and a thickness of about 6.5 mm were utilized. The molding process was conducted using a laboratory setup at mcd with dedicated process control. Prior to resin monomer deposition, the glass surface was treated with an adhesion promoter. The commercially available resin Ormocomp^®^ (micro resist technology GmbH, Berlin, Germany) served as the base material. A planar stamp functioned as the upper mold, and under applied pressure and exposure to 365 nm UV light at room temperature and 50% relative humidity, the hybrid polymer was cured. Upon completion, the stamp was removed, yielding a polymer layer approximately 20 µm thick. In this study, only a circular region with a 10 mm diameter was coated, leaving the remaining substrate untreated. Key advantages of the UV molding process for hybrid polymers include high precision and surface quality, the ability to form complex geometries and freeform optics, and cost efficiency. Furthermore, UV molding offers scalability, fast curing times, and the possibility of low processing temperatures. State-of-the-art research demonstrates that this technique facilitates the fabrication of large-area nanostructures, with patterned surfaces ranging from a few square centimeters to sizes up to 200 mm, extending to display-scale dimensions [24].

Reactive ion etching (RIE) using an Oxford Plasmalab 800 system was employed to create nanopillars on the substrates. The process involved two sequential etching steps to control the nanopillar geometry and refractive index profile. The first step utilized a 1:1 mixture of Ar and SF_6_ (60 sccm, 50 mTorr, RF power 500 W, 60 s) followed by a 1:1 mixture of Ar and CHF_3_ (60 sccm, 50 mTorr, RF power 350 W, 20 s). These steps were repeated in carefully optimized cycles until the desired structure depth was achieved, with the sample temperature maintained at 20 °C throughout the process. To derive the optimal parameters, we started from standard etching processes for inorganic fused glass and optical polymers and systematically varied process parameters such as gas flow, pressure, and RF power, using transmittance and reflectance measurements as assessment criteria.

**Figure 1:**
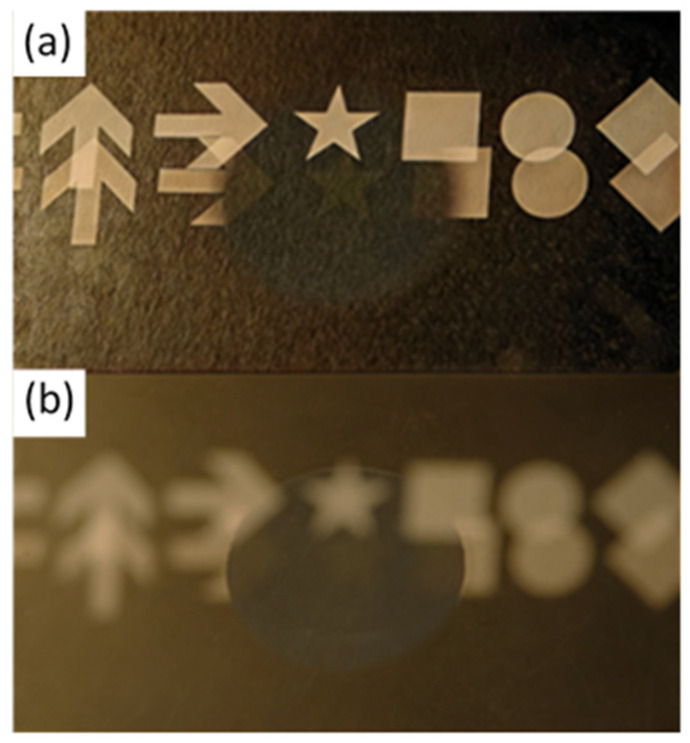
Photographs of reflections on a substrate, where the central region of the front surface is equipped with AR structures. Figure (**a**,**b**) show different focus settings. In the outer regions, two reflections can be observed, caused by reflections from the front and back surfaces of the substrate. In the central region, only the reflection from the back surface is visible due to the antireflective structuring on the front surface.

To visualize the effect of moth-eye-structured surfaces on hybrid polymer materials, Figure 1 shows photographs comparing AR-coated regions with untreated areas. The previously prepared glass substrate, with the hybrid polymer applied to its central circular area and the AR structures confined to this region, was used. The images capture reflections of a scene consisting of simple symbols such as arrows, squares, circles, and stars. In the top image, the camera was focused on the scene’s distance, rendering the symbols sharp. In the bottom image, the camera was focused on the substrate surface, making the circular transition between the polymer-coated and polymer-free areas appear sharp while blurring the background symbols. Double reflections from the front and back surfaces of the substrate are visible in the outer regions, whereas only the back-surface reflection is observed in the central area due to the antireflective structuring. For example, the central star appears only once, demonstrating the AR effectiveness in the structured region.

High-magnification scanning electron microscopy (SEM) imaging was challenging due to sample charging. The sample surface was coated with a thin titanium layer by sputtering using a magnetron coater. In contrast to alternative coating materials, titanium coating may slightly alter surface structures; however, it enhances beam stability and minimizes polymer degradation. Additionally, conductive silver was applied from the edge to the center to further reduce charging effects.

SEM imaging and focused ion beam (FIB) cross-sectioning were performed using a dual-beam microscope. For FIB, Gallium (Ga^+^) ion beam sputtering at 30 kV was conducted without a protective pad. This was due to the presence of high-aspect-ratio structures, which made the deposition of a homogeneous pad challenging, and because the pad would have partially covered the view of the surface morphology. A low ion current of 10 pA was used to minimize material modification. Imaging at 4.5 kV balanced charging effects and beam-induced degradation, as lower voltages resulted in image drift. To optimize the signal-to-noise ratio, micrograph acquisition incorporated short pixel dwell times, frame averaging, and drift correction. Image stabilization was further improved by allowing surface exposure prior to capture, ensuring high-quality, high-magnification micrographs.

**Figure 2:**
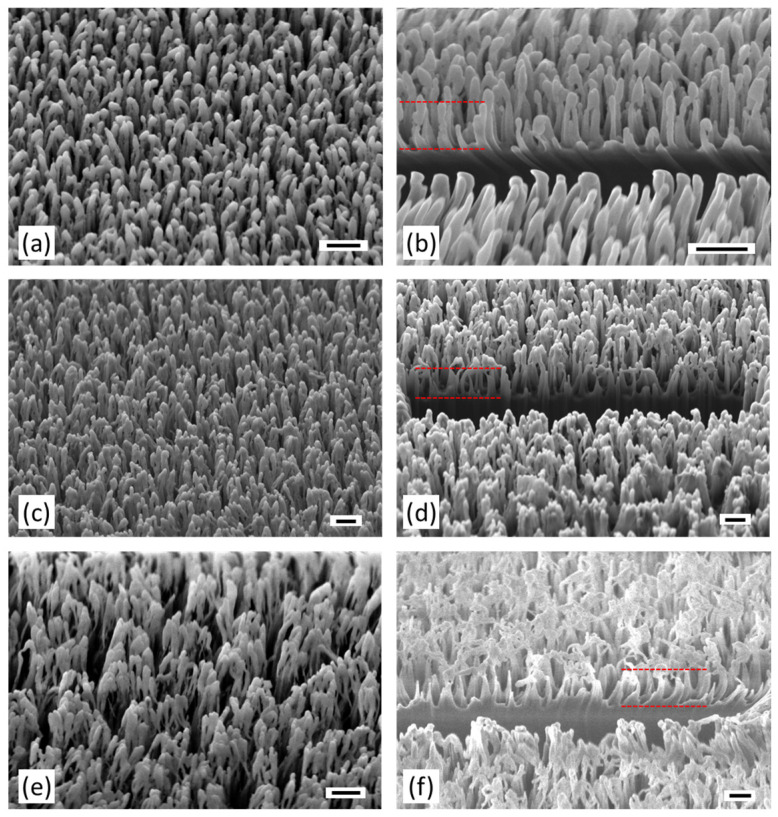
SEM images of stochastically distributed, pillar-like antireflective “moth-eye” structures in hybrid polymers fabricated via an RIE process. Rows correspond to different etching durations and resulting pillar depths. Left column (**a**,**c**,**e**): surface overviews; right column (**b**,**d**,**f**): FIB-generated cross sections. Red dashed lines indicate average pillar heights of 450 nm, 725 nm, and 830 nm. Images were recorded at an oblique angle of 54°. Scale bar: 400 nm.

Using these optimized imaging parameters, high-quality SEM images were obtained, as shown in Figure 2, to analyze the effect of different etch times on the resulting structures. The scale bar in all images corresponds to 400 nm, and all were recorded at an oblique angle of 54°. Each row represents results for a specific etching duration: the first row (Figure 2a,b) corresponds to 320 s, the second row (Figure 2c,d) to 480 s, and the third row (Figure 2e,f) to 560 s. The left column displays unmodified surface overviews, revealing densely packed moth-eye structures uniformly covering the surface. In the longest etching time sample (Figure 2e), freestanding pillars appear to attract each other at their upper ends, forming pillar bundles with larger statistical periods or lower spatial frequencies that may lead to parasitic scattering effects rather than optimal AR performance.

The right column shows the corresponding images, where the FIB was used to create a cross section in the near-surface region of each sample. The FIB cut provides a direct view into the 3D structure of the pillars, enabling the determination of the geometrical dimensions of the moth-eye structures. Despite etching duration variations, statistical pillar distances remain approximately 200 nm. However, pillar heights increased with etching time, with average heights of 450 nm, 725 nm, and 830 nm corresponding to increasing etching durations.

It can be observed that the increase in etching time does not correlate linearly with the achieved average pillar heights. Furthermore, as can be clearly seen in Figure 2f, the surface, especially in areas behind the FIB cut, shows deposits. This suggests that the upper parts of the pillars have broken off and are now partially dispersed over the surface. In summary, it seems that with increasing etching time, the growth of the structural depth is even accelerated. Finally, it is noteworthy that the target depth of moth-eye structures used for antireflection purposes lies in the range of $\frac{\lambda }{4*n_{mean}}$ (*n_mean_*: mean refractive index of moth-eye structure layer) [25]. This implies that depths of approximately 400 nm are sufficient for antireflection of wavelengths up to 2 µm.

It should be noted that a key advantage of the proposed method is that the antireflective structures are applied only after the printing process and are therefore not influenced by UV molding. At the same time, the wavefront quality achieved through the UV molding process remains unaffected by the precise etching process.

## 3. Angle-Resolved Specular Transmission and Reflection Measurements

To validate the antireflective properties of the etched Ormocomp^®^ samples, a spectrophotometer (Lambda 1050+; PerkinElmer, Tempe, AZ, USA) equipped with a Total Absolute Measurement System (TAMS) kit was utilized. The measurements were performed on samples with different average pillar heights (450 nm, 725 nm, and 830 nm) over a wavelength range from 250 nm to 2000 nm in steps of 10 nm. Both transmission and reflection measurements were performed using two different setups, varying the angle of incidence from 0° to 70° in 10° increments. An unstructured Ormocomp^®^ sample served as a reference to illustrate the advantages of the structured samples. Baseline measurements were recorded in air for all experiments, with the angle of incidence defined as the angular relationship between the incident light and the horizontal plane. The data uncertainty is slightly above 0.2%, which is consistent with the specifications provided by the manufacturer of the spectrophotometer.

### 3.1. Angle-Dependent Specular Transmittance

In the first step, transmission measurements were carried out for varying incidence angles. The TAMS setup enabled automated adjustment of both the sample angle and detector position. Samples were mounted on a rotationally adjustable platform while the detector recorded the transmitted light across different wavelength ranges.

**Figure 3:**
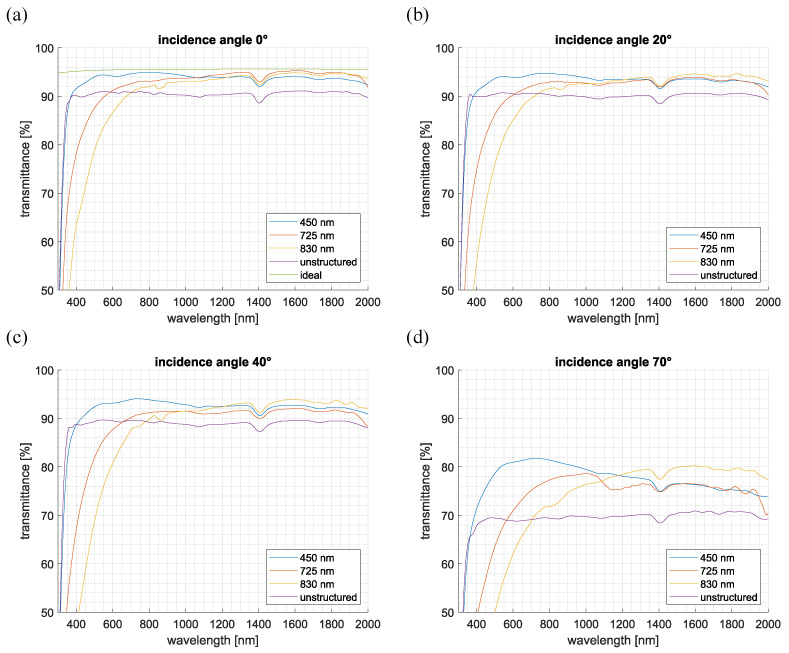
Transmission curves of the unstructured Ormocomp^®^ layer and the structured layers with average pillar heights of 450 nm, 725 nm, and 830 nm at angles of incidence of 0° (**a**), 20° (**b**), 40° (**c**), and 70° (**d**). (**a**) “Ideal” transmission curve (green) as a reference, compensating for Fresnel reflections on the backside and taking into account the dispersion curves of the involved materials.

Figure 3 presents the measurement results of specular transmission for the moth-eye-structured Ormocomp^®^ samples as well as for the unstructured reference sample. For clarity, only results for incidence angles of 0° (a), 20° (b), 40° (c), and 70° (d) are displayed. To better assess the performance of the AR structures and, in particular, to compensate for the always present back-reflection, an “ideal” transmission curve (green) was additionally plotted as a further reference curve in Figure 3a (0°; normal incidence). This ideal curve assumes complete transmission through the AR structure and considers all further Fresnel reflections at each interface as well as the quantitative values of the dispersion curves for fused silica [26] and Ormocomp^®^ [27].

The unstructured sample showed a transmission that increased sharply from 300 nm to 380 nm, reaching a maximum of 91%, which remained nearly constant for longer wavelengths. In contrast, the different AR-structured samples exhibited varying transmission behaviors, with the transition to very high transmission values (transmittance > 90%) occurring at different wavelength ranges depending on the pillar height. In particular, the sample with a pillar height of 450 nm reached high transmission values, similar to the unstructured sample, already at around 360 nm, while the samples with average heights of 725 nm and 830 nm only reached high transmission values at wavelengths of 590 nm and 700 nm, respectively. These spectral shifts in the transition range to high transmission values may be caused by the bundling of the pillars or the potential deposition of broken pillar tips, which introduce subperiodic structures, leading to increased scattering and reduced transmission at shorter wavelengths. Despite these differences, all structured samples achieved very high maximum transmission values (94–95%), very close the theoretical maximum transmission of the ideal curve at 95.6%. The transmission curves reveal that shorter pillar heights enhance transmission at shorter wavelengths, whereas taller pillars are more effective for longer wavelengths. This trend is also confirmed by the comparison with the ideal curve. Specifically, the curve for the sample with an average pillar height of 450 nm reaches the values of the ideal curve at 800 nm. For the curves of the samples with average pillar heights of 725 nm and 830 nm, the ideal curve is reached at a wavelength of approximately 1600 nm.

As the angle of incidence increased, the qualitative trends remained unchanged for both structured and unstructured samples. Although the transmission values decreased with the increasing angle, the spectral positions of the key features remained relatively constant. At an incidence angle of 70°, the maximum transmission efficiency for the unstructured sample dropped to 69%, while the structured samples maintained values between 79% and 82%. This increase of over 10% confirms the significantly improved transmittance of the structured samples.

### 3.2. Angle-Dependent Specular Reflectance

**Figure 4:**
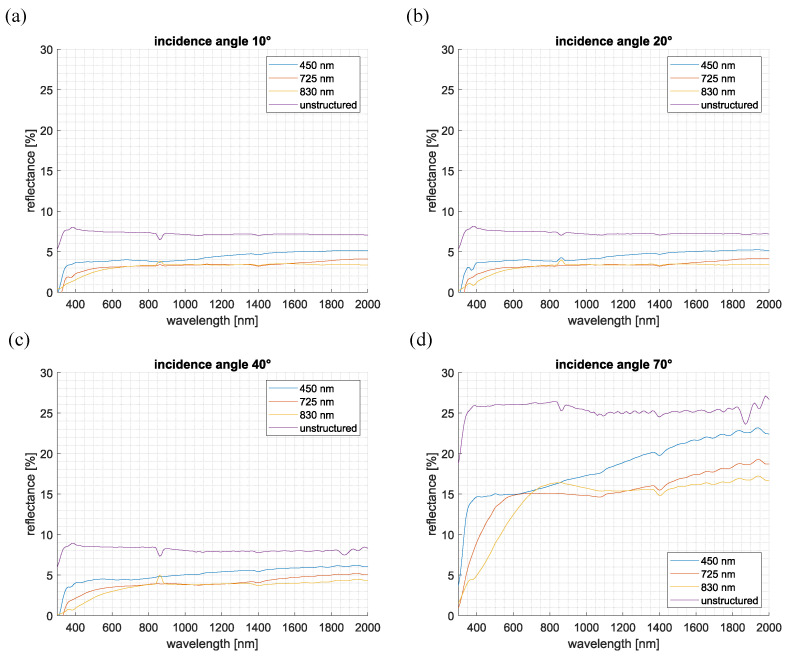
Reflection curves of the unstructured Ormocomp^®^ layer and the structured layers with average pillar heights of 450 nm, 725 nm, and 830 nm at angles of incidence of 10° (**a**), 20° (**b**), 40° (**c**), and 70° (**d**).

For completeness, specular reflection measurements on the samples were subsequently performed at various angles of incidence. Utilizing the TAMS setup, the reflected light was collected by an integrating sphere while the detector angle was automatically adjusted. Measurements were performed on both structured and unstructured samples for reference. The results are presented in Figure 4 for incidence angles of 10°, 20°, 40°, and 70°.

At normal incidence (0°), the unstructured sample displayed a maximum reflectance of 8% at 380 nm, gradually decreasing to 7% at 2000 nm. In contrast, the AR-structured samples exhibited significantly lower reflectance across the entire usable wavelength range. The reflectance minimum occurred at shorter wavelengths and gradually increased toward longer wavelengths. It can be observed that reflection suppression improves with increasing pillar height. The sample with an average pillar height of 830 nm reached a minimum reflectance of 3.4% at 2000 nm, compared to 5.1% for samples with an average pillar height of 450 nm. The observation that taller pillar structures lead to reduced reflection at shorter wavelengths, combined with transmission measurements showing decreased light transmittance in this wavelength range, suggests that unwanted light scattering occurs. These scattering effects may originate, as previously mentioned, from the introduction of substructures caused by the bundling of the pillars or from structural defects resulting from the potential deposition of broken pillar tips.

For incidence angles up to 40°, the specular reflectance curves for all samples exhibited minimal variation in absolute values. However, at higher incidence angles, a significant increase in reflectance was observed. At 70°, the unstructured sample exhibited a maximum reflectance of 26%, while the structured samples showed values ranging between 17% and 23%. Despite the variations in absolute reflectance, the qualitative behavior and relative ordering of the curves remained consistent across all incidence angles.

**Figure 5:**
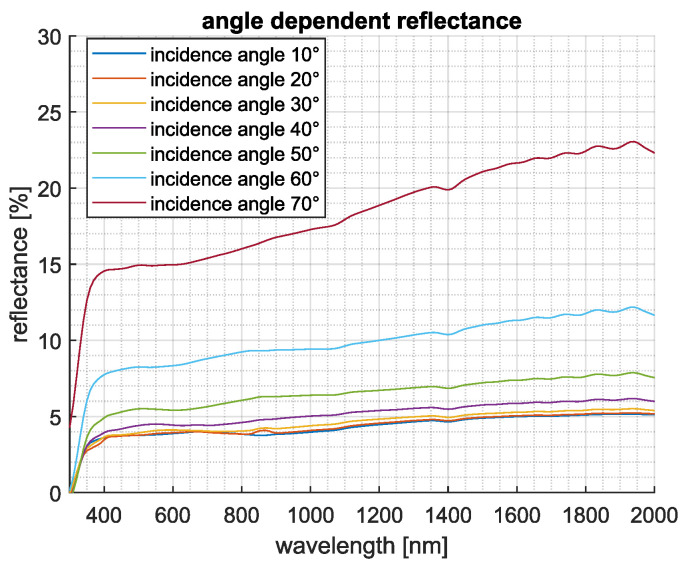
Reflectance curves of the structured layer with an average pillar height of 450 nm at angles of incidence from 10° to 70° in 10° steps.

For completeness, Figure 5 presents the reflectance of the structured sample with an average pillar height of 450 nm across all measured incidence angles from 10° to 70°. The data clearly indicate that for incidence angles below 40°, no substantial increase in reflectance occurs. Even at 70°, the maximum reflectance remains below 23%, further highlighting the effectiveness of the structured surface in reducing optical losses at oblique angles.

## 4. Hydrophobicity and Thermal Stability

The practical suitability of AR-structured Ormocomp^®^ as a lens material for optical systems depends not only on its direct optical properties but also on its stability against environmental influences and on desirable properties such as dirt- or water-repellent characteristics. For this reason, we also included the wetting properties of the samples and their thermal stability in our investigations.

**Figure 6:**
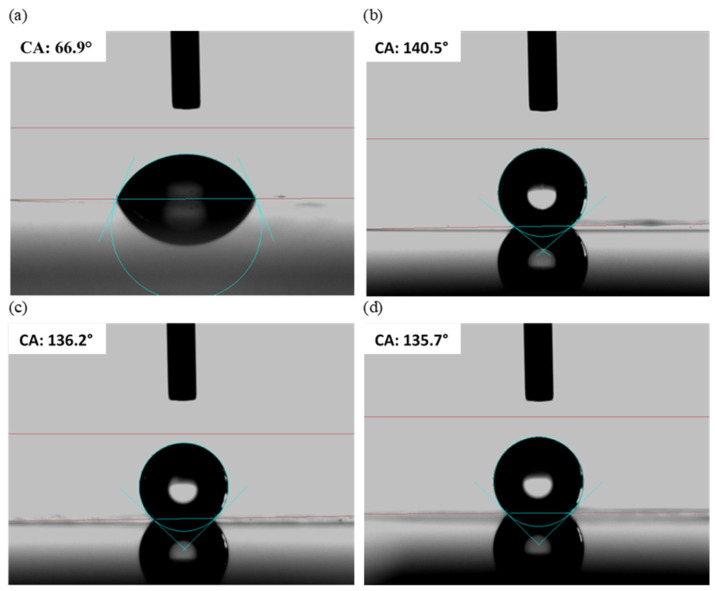
Contact angles of (**a**) the unstructured Ormocomp^®^ layer and the structured layers with average pillar heights of 450 nm (**b**), 725 nm (**c**), and 830 nm (**d**).

The wetting properties were assessed through contact angle measurements using distilled water. Figure 6 displays the results of the contact angle (CA) measurements for an unstructured Ormocomp^®^ layer (a) and structured layers with pillar heights of 450 nm (b), 725 nm (c), and 830 nm (d). Since the measured contact angles of the droplet applied to the sample surface were nearly identical on the left and right sides, only the measurements from the right side were considered.

The unstructured Ormocomp^®^ surface exhibited a contact angle of 69.9°, whereas the structured samples demonstrated significantly higher values, more than double that of the unstructured layer (structure height → contact angle: 450 nm → 140.4°; 725 nm → 136.2°; 830 nm → 135.7°). This significant increase in the contact angle highlights the enhanced resistance of the AR-structured surfaces to water-based contamination.

The slight decrease in the contact angle for samples with a higher average pillar height (increase in pillar height from 450 nm (b) to 830 nm (d)) is likely due to the formation of surface defects, which could be caused by the deposition of broken pillar tips. The pronounced water-repellent properties of the structured Ormocomp^®^ layers are attributed to the significant increase in the substrate surface area due to the nanoscale surface architecture.

To investigate the thermal stability, a sample with an average pillar height of 450 nm was selected. This sample was subjected to controlled thermal loads at different temperatures, and its transmission and reflection values were subsequently measured. Specifically, the sample was heated in an oven from 50 °C to 350 °C in 50 °C intervals, with each temperature held for 30 min. After each heating step, transmission and reflection measurements were taken again at room temperature using a Perkin Elmer Lambda 1050+ spectrophotometer following the procedure described in the previous section. Transmission measurements were conducted at normal incidence (0°), while reflectance was measured at a 10° angle of incidence.

**Figure 7:**
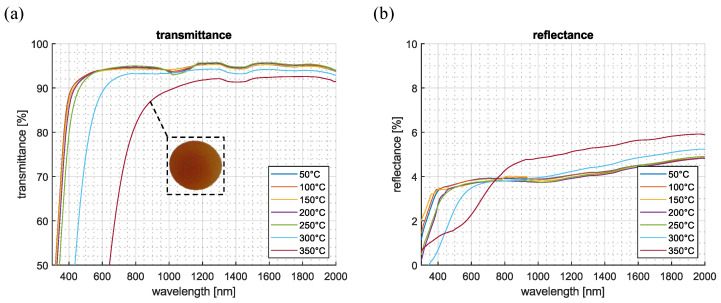
(**a**) Transmittance and (**b**) reflectance spectra of structured Ormocomp^®^ layer with an average pillar height of 450 nm at different temperatures when the light incident angle is 0° for transmission and 10° for reflection. The dashed square in (**a**) shows the yellowing of the Ormocomp^®^ layer observed after exposure to 350 °C.

The results shown in Figure 7 indicate that temperatures below 250 °C had no significant effect on either transmittance or reflectance. The optical properties remained stable, confirming the structural integrity of the moth-eye nanostructures within this temperature range. However, at temperatures above 250 °C, transmittance began to decline, accompanied by a shift in the absorption threshold toward longer wavelengths, resulting in a visually noticeable yellowing effect, also shown in Figure 7a in the dashed square. Despite this change in the short-wavelength range, the AR-structured sample maintained higher transmittance than the unstructured sample at wavelengths beyond 1200 nm. This suggests that the nanostructures remained intact and that absorption losses were primarily due to modifications in the bulk material rather than structural degradation.

Similarly, reflectance remained nearly unchanged for temperatures up to 250 °C, confirming the stability of the moth-eye antireflective structures within this range. At 300 °C and 350 °C, a gradual increase in reflectance was observed. However, reflectance values remained lower than those of the unstructured sample. This indicates that the antireflective performance, although slightly diminished, remained effective. Overall, these findings demonstrate that the structured Ormocomp^®^ layers exhibit enhanced environmental resistance, providing both hydrophobic properties and thermal stability up to 350 °C while effectively reducing optical losses.

## 5. Conclusions

This study demonstrates that the surfaces of hybrid polymers, which combine the advantageous properties of inorganic glasses and organic polymers, can be equipped with antireflective (AR) structures through reactive ion etching (RIE). Without requiring additional layer preparation, deep, statistically distributed pillar-like structures with subwavelength spacing can be generated. By adjusting the etching time, the pillar heights can be precisely controlled, with average heights of several hundred nanometers enabling effective antireflection across the visible-to-infrared spectral range. The structured samples exhibited significant optical performance enhancements over a broad wavelength range from 450 nm to 2 µm. The transmittance increased by more than 4% per interface, approaching the theoretical maximum.

The exceptional thermal stability of these AR structures is further demonstrated by their unaltered optical performance following prolonged exposure to temperatures up to 250 °C. Specifically, no significant changes in transmission or reflection behavior were observed. However, exposure to even higher temperatures resulted in the occurrence of a yellowing effect. This effect indicates substantial alterations in the bulk properties of the hybrid polymer, whereas changes in AR properties remain secondary. In addition to their optical benefits, the fabricated AR structures exhibit advantageous hydrophobic properties, as verified through contact angle measurements.

The proposed method for equipping optical components made of hybrid polymers with AR structures appears promising for commercial applications. Potential use cases include the automotive industry, where components are exposed to high temperatures. Moreover, the process costs seem suitable for this application field. For instance, the initial investment in etching equipment is estimated to be comparable to that of coating systems typically used for antireflection purposes. Additionally, the production times and processable batch sizes of the RIE process are similar to those of traditional layer coatings.

## Data Availability

The original contributions presented in this study are included in the article.
